# Imprecise prediction of implant sizes with preoperative 2D digital templating in total knee arthroplasty

**DOI:** 10.1007/s00402-023-04772-7

**Published:** 2023-01-17

**Authors:** Felix Riechelmann, H. Lettner, R. Mayr, R. Tandogan, D. Dammerer, M. Liebensteiner

**Affiliations:** 1 Department of Orthopaedics and Traumatology, Medical University of Innsbruck, Anichstrasse 35, 6020 Innsbruck, Austria; 2 Medical University of Innsbruck, Anichstrasse 35, 6020 Innsbruck, Austria; 3 Ortoklinik, Ankara, Turkey; 4 Department of Orthopaedics and Traumatology, Halic University, Istanbul, Turkey; 5 University Hospital Krems, Krems, Austria

**Keywords:** Total knee arthroplasty, Digital templating, Accuracy, EndoCert, Operative time, Storage cost

## Abstract

**Purpose:**

To analyze the match between preoperatively determined implant size (2D templating) and intraoperatively used implant size in total knee arthroplasty (TKA). Also examined were the factors that might influence templating accuracy (gender, surgeon experience, obesity, etc.).

**Materials and methods:**

The study was retrospective and conducted in a specialized ENDOCERT arthroplasty center. Digital templating was done with the MediCAD software. If the planned and implanted TKA components (both femur and tibia) were the same size, the match was classified “exact.” A deviation of ± one size (at the femur or tibia or both) was classified “accurate.” A deviation of ± two or more sizes (at the femur or tibia or both) was classified “inaccurate.” Obesity, gender, implant type and surgeon experience were investigated for potential influence on templating accuracy. Chi-square tests and Cohen’s weighted kappa test were used for statistical analysis.

**Results:**

A total of 482 cases [33.6% male, 66.4% female, age 69 ± 11, body mass index (BMI) 30.3 ± 5.8] were included. When the femur and tibia were taken together, exact size match was observed in 34% (95% CI 29.9–38.3%) of cases, accurate size match in 57.5% (95% CI 53–61.8%) and inaccurate size match in 8.5% (95% CI 6.3–11.2%). Inaccurate size match prolonged operative time (*p* = 0.028). Regarding the factors potentially influencing templating accuracy, only gender had a significant influence, with templating being more accurate in men (*p* = 0.004). BMI had no influence on accuracy (*p* = 0.87). No effect on accuracy was observed for implant type and surgeon experience.

**Conclusions:**

The accuracy of 2D size templating in TKA is low, even in a specialized ENDOCERT arthroplasty center. The study findings challenge the usefulness of preoperative 2D size templating and highlight the importance of more reliable templating methods.

**Level of evidence:**

Level III (retrospective observational study).

## Introduction

Preoperative radiographic planning is an essential factor for better clinical and functional outcomes following total knee arthroplasty (TKA) [1]. It allows the mechanical and anatomical axes of the leg, implant sizes and optimal implant position to be estimated. Bone deformities and defects are identified preoperatively so that necessary modifications to the standard surgical procedure can be made. In addition, preoperative radiographic planning allows the appropriate surgical instrumentation to be provided [2]. In recent years, many centers have switched from acetate templating to 2D digital templating for TKA. Results of digital templating were more accurate as they were less affected by magnification errors. However, prediction of implant size still varied and perfect prediction could not be achieved [3–5]. Recently, 3D templating, demographic variables and mathematical equations were introduced to optimize preoperative size prediction, but still without perfect results [6, 7].

The actual implant size is determined by the surgeon intraoperatively. If the implant size is not successfully predicted preoperatively (e.g., with 2D templating), a large range of implants have to be held in stock. As the range of implant sizes and shapes is constantly growing to account for different patient ethnicities [8] and gender [9, 10], storage capacity has to grow as well. This leads to the need for larger storage rooms and thus increased cost. If preoperative templating is exact, only one component size must be available in the operating theater. To compensate an error of ± one size of the planned implant, six components must be available in or close to the operating theater during surgery. Larger deviations from the planned size usually require the assistant to fetch the needed implant from storage. All these factors lead to increasing costs for stock, preparation time and operating time [11]. To reduce the number of needed component sizes in stock and in or close to the operating theater, accurate size predictions are needed. However, current data on prediction accuracy are sparse.

In this study, we aimed to analyze the current accuracy of 2D digital size templating in a certified arthroplasty center (EndoCert EPCmax). EndoCert is the world’s first certification system in arthroplasty. It aims to certify medical facilities for joint replacement by the German Society for Orthopedics and Orthopedic Surgery, the Working Group Endoprosthetics (AE) and the Association of Orthopedics and Trauma Surgery (BVOU). Medical facilities can be certified through audits as an Endoprosthetics Center (EPC) or as a Maximum Care Endoprosthetics Center (EPCmax).

The purpose of this study was to determine the accuracy of 2D digital templating in predicting femoral and tibial implant sizes at a Maximum Care Endoprosthetics Center and to determine which factors might influence 2D digital templating accuracy.

## Materials and methods

All patients who underwent primary TKA between January 2017 and August 2019 at the Department of Orthopaedics and Traumatology were included. 2D digital templating was performed in all patients. Implant types evaluated were the Attune system (Depuy-Synthes, Raynham, MA, USA) and the Triathlon system (Stryker, Kalamazoo, MI, USA), which were the two major systems used. Available sizes for the Depuy-Synthes Attune system ranged from one to ten and for the Stryker Triathlon from one to eight. Both cruciate-retaining (CR) and posterior-stabilized (PS) implants were analyzed. Patients with revision TKA, primary TKA with revision implants or patients without preoperative 2D digital templating were not considered for inclusion. The procedures used complied with the Declaration of Helsinki of 1975 and its revision of 1983. The ethical, legal and regulatory norms and standards for research involving human subjects as well as the relevant international norms and standards were taken into account. Approval was obtained from the Ethics Committee of the Medical University of Innsbruck (reference number 1150/2019).

All radiographs were taken with the same technique by the Department of Radiology of blinded institution. The projections were true antero-posterior, true lateral and long-leg axis. The tube-to-film distance was standardized at 1.15 m. A radiopaque ball with a diameter of 25 mm served as size reference. For quality control, a radiologist and an orthopedic surgeon checked appropriate alignment of radiographs in two planes (true anterior–posterior/lateral). Inappropriate radiographs were repeated. Preoperative digital templating was performed with MediCAD (MediCAD Hectec GmbH, Altdorf/Landshut, Germany). Size templating had been stored in the PACS (Picture Archiving and Communication System) for the femoral and tibial component in both lateral and AP images. Lateral femoral size templating and the AP tibial size templating were taken into consideration and then compared with the surgical reports. Implant size predicted with 2D templating was compared with the actual implant size as determined in the surgical report. Additionally, data including age, gender, side of operation, operative time and BMI were obtained with the clinical information system. The surgeons’ experience was categorized as resident surgeon, specialist with =  < 4 years of experience and senior specialist with > 4 years of experience.

Frequency data were tabulated and analyzed with Chi-square tests. Depending on distribution, means and standard deviations or medians and upper and lower quartiles were calculated for interval-scaled variables. Cohen’s weighted kappa with quadratic weighting was used as a measure of concordance between planned and implanted TKA components. If the planned and implanted TKA components (both femur and tibia) were the same size, the match was classified “exact.” A deviation of ± one size (at the femur or tibia or both) was classified as “accurate.” A deviation of ± two or more sizes (at the femur or tibia or both) was classified as “inaccurate.” Whether interval-scaled data differed as a function of matching accuracy was tested using analysis of variance. The 95% confidence limits of percentages were calculated using SPSS custom tables (SPSS 26, IBM Corporation, Armonk, NY, USA). BMI was classified according to the WHO classification; adiposity was defined as a BMI ≥ 30 (obesity).

## Results

**Fig. 1:**
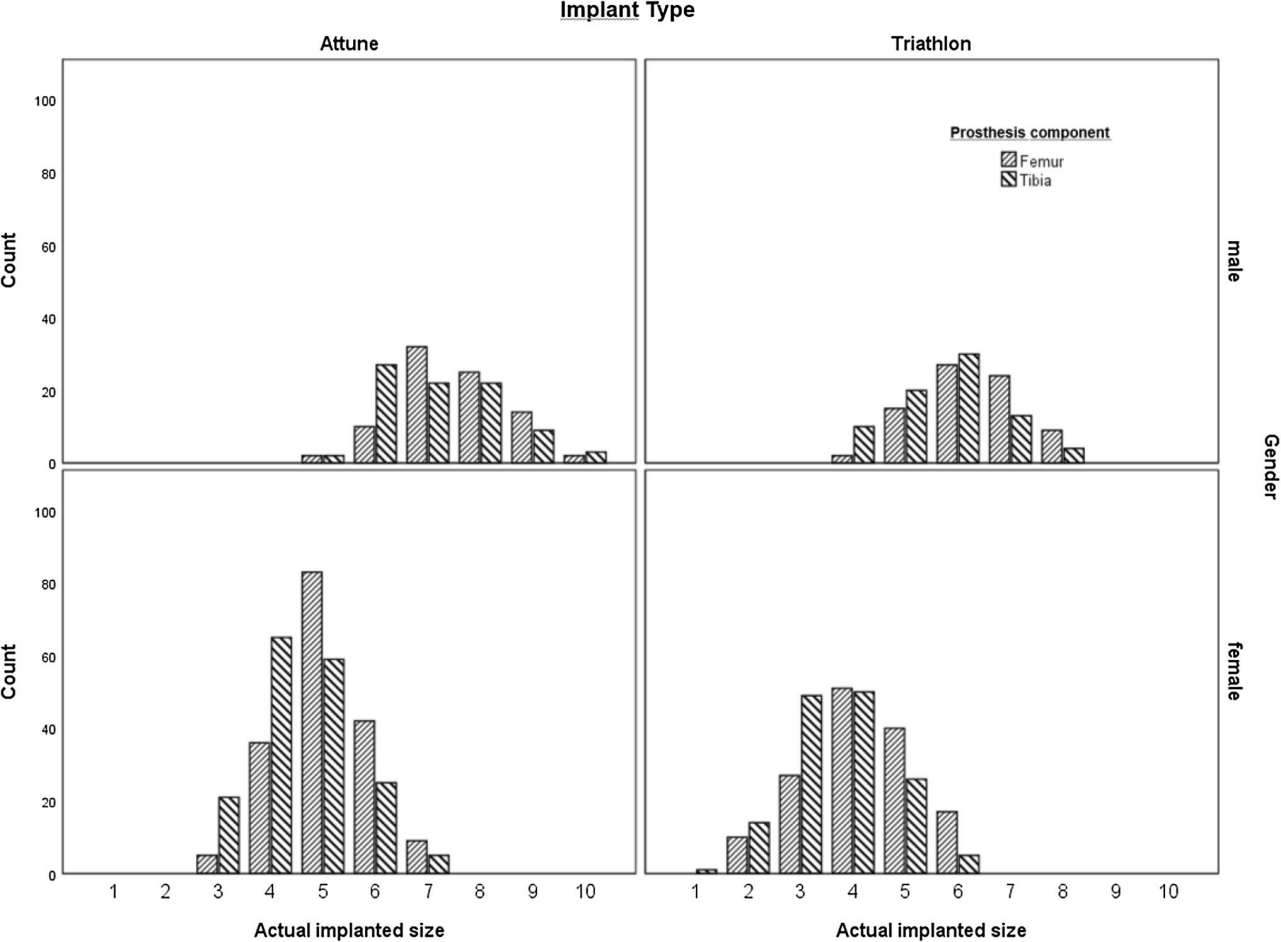
Actual implanted Attune (size range 1–10) and Triathlon (size range 1–8) total knee prosthesis sizes in 162 male (upper panel) and 320 female (lower panel) patients

A total of 482 total knee arthroplasties were performed in patients who met the inclusion and exclusion criteria. Mean (± SD) patient age was 69 ± 11 years. Mean BMI was 30.3 ± 5.8. Additional patient characteristics are listed in Table 1. An overview of the implants used is provided in Fig. 1.

**Table 1:** Patients and treatment characteristics for 482 total knee arthroplasties

	Count	Column *N* %
*Gender*		
Male	162	33.6
Female	320	66.4
*Side*		
Left	218	45.2
Right	264	54.8
*Implant type*^a^		
Attune	260	53.9
Triathlon	222	46.1
*Model*		
CR	283	58.7
PS	199	41.3
*Surgeon experience*		
Senior specialist > 4 years	265	55.0
Specialist = < 4 years	146	30.3
Resident surgeon	71	14.7
*WHO BMI class*		
< 18.5	5	1.0
18.5–24.9	72	14.9
25.0–29.9	190	39.4
30.0–34.9	114	23.7
35.0–39.9	67	13.9
40.0 +	34	7.1
*Adiposity*		
No adiposity	267	55.4
Adiposity	215	44.6

*CR* cruciate-retaining, *PS* posterior-stabilized

^a^Attune system (Depuy-Synthes, Raynham, MA, USA) and Triathlon system (Stryker, Kalamazoo, MI, USA)

When the femur and tibia are taken together, exact matching was observed in 34% of all total knee arthroplasties, accurate matching in 57.5% and inaccurate matching in 8.5%. When considering the femur and tibia separately, exact matching on the femoral side was found in 266 (55.2%) implants. On the tibial side, exact matching was found in 255 (52.9%; Table 2) knees. Cohen’s weighted kappa as a measure of concordance between planned and implanted size was 0.88 (95% CI 0.86 to 0.91; *p* < 0.001) at both the femur and the tibia.

**Table 2:** Observed planning accuracy of 2D digital size templating at both femur and tibia (total) and at femur and tibia separately in 482 total knee arthroplasties

	Count	Percent	95.0% lower CL^a^	95.0% upper CL
*Planning accuracy total*				
Exact	164	34.0	29.9	38.3
Accurate (± 1 size)	277	57.5	53.0	61.8
Exact or accurate^b^	441	91.5	82.9	100.1
Inaccurate (± 2 sizes or worse)	41	8.5	6.3	11.2
*Planning accuracy femur*				
Exact	266	55.2	50.7	59.6
Accurate (± 1 size)	199	41.3	37.0	45.7
Exact or accurate	465	96.5	87.7	105.3
Inaccurate (± 2 sizes or worse)	17	3.5	2.1	5.5
*Planning accuracy tibia*				
Exact	255	52.9	48.4	57.3
Accurate (± 1 size)	198	41.1	36.8	45.5
Exact or accurate	453	94.0	85.2	102.8
Inaccurate (± 2 sizes or worse)	29	6.0	4.2	8.4

^a^Confidence limit for column percent

^b^For easier comparison with Table 3

The only patient factor that significantly affected templating accuracy was gender. Exact matches were observed in 43.8% of males and 29.1% of females (*p* = 0.004). No significant influence was observed for age (*p* = 0.96) or BMI (*p* = 0.87). Surgeon experience also had no significant effect on planning accuracy (*p* = 0.58). No significant difference in planning accuracy was observed between the two types of implant used (Attune and Triathlon; *p* = 0.16).

**Fig. 2:**
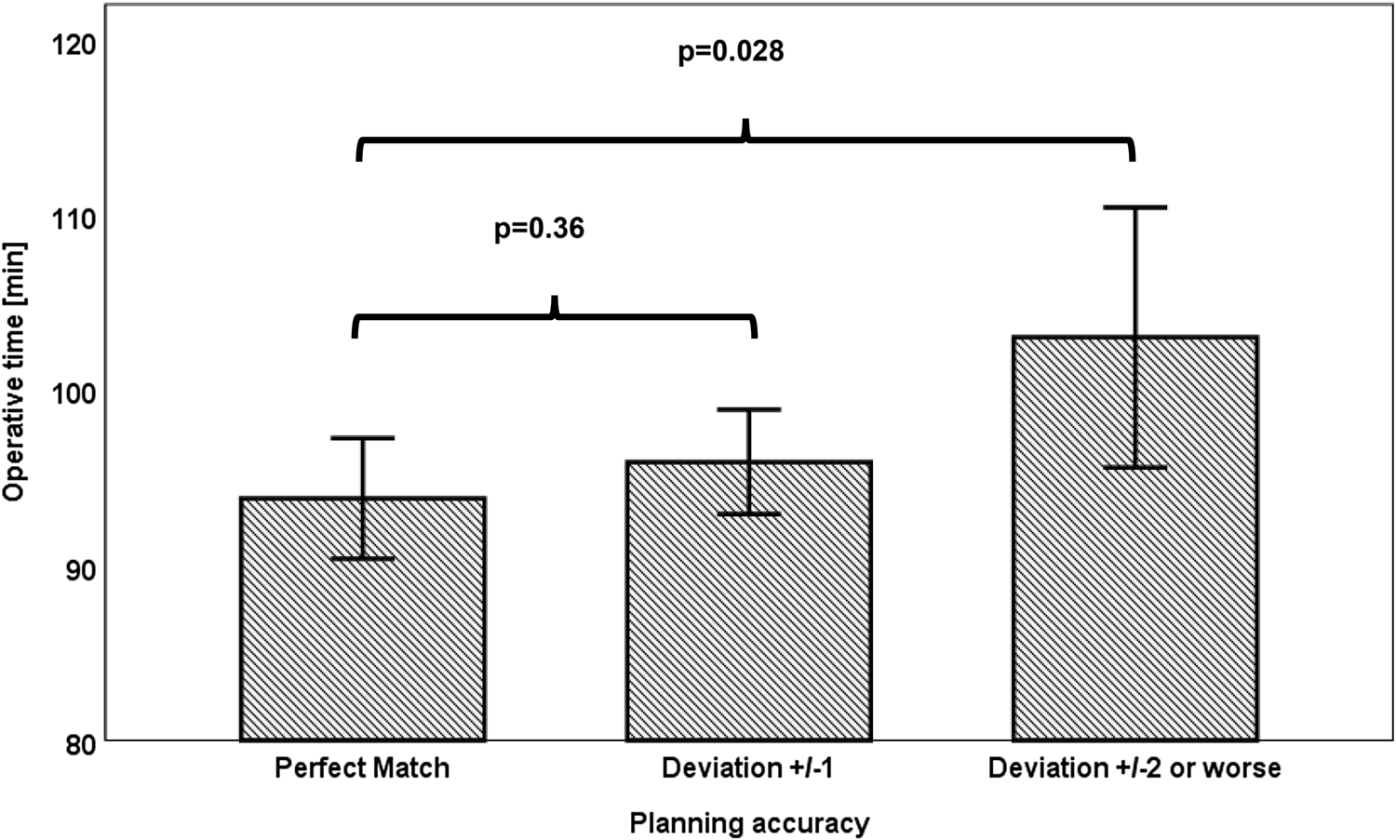
Mean operative time in minutes in TKA with perfect match (*n* = 164), accurate match (*n* = 277) and inaccurate match (*n* = 41) corrected for gender and adiposity. Error bars indicate 95% confidence intervals

Mean operative time was influenced by 2D templating accuracy. After correction for sex and adiposity, the mean operative time for cases templated “exact” was 93.8 ± 1.8 min, for cases templated “accurate” (± one size) it was 95.9 ± 1.5 min (*p* = 0.3), and for cases templated “inaccurate” (± two sizes or worse) it was 103.1 ± 3.8 min (*p* = 0.028) (Fig. 2).

## Discussion

The most important finding of the current study was that 2D digital templating of total knee implants resulted in exact matches for both femur and tibia in only 34% of the nearly 500 total knee arthroplasties investigated. The results of this study are in line with previous reports on 2D planning accuracy in total knee arthroplasty (Table 3). Of all the factors studied, including age, gender, BMI and surgeon experience, only gender showed a significant effect on 2D templating accuracy, with the templating being more accurate in men.

**Table 3:** Reported accuracy of 2D and 3D templating in total knee arthroplasty

Authors	Year	*N* =	Method	Accuracy overall %	Exact match femur %	Exact match tibia %	Exact or accurate match femur %	Exact or accurate match tibia %
Kniesel et al. [12]	2014	46	2D templating with calibration marker	n.a.	n.a.	n.a.	98	100
Miller et al. [13]	2012	50	2D templating with 115% magnification	n.a.	65	60	100	100
Hernandez-Vaquero et al. [14]	2013	50	2D templating, constant distance knee to X-ray tube	n.a.	55	50	90	94
Wallace et al. [15]	2020	181	2D templating, inconsistent use of reference markers	n.a.	35.4	36.5	86.2	85.1
Seaver et al. [16]	2020	125	2D templating, manual planning	n.a.	56	61.6	97.6	95.2
León-Muñoz et al. [17]	2020	336	3D templating	n.a.	95.8	92.6	100	100
Pietrzak et al. [18]	2019	31	2D templating	n.a.	52.9	28.7	n.a.	n.a.
Pietrzak et al. [18]	2019	31	3D templating	n.a.	96.6	93.1	n.a.	n.a.
Kobayashi et al. [19]	2012	100	3D templating	98	n.a.	n.a.	n.a.	n.a.
Kobayashi et al. [19]	2012	100	2D templating	59	n.a.	n.a.	n.a.	n.a.

*n.a.* not available

In the studies cited in Table 3, a major factor affecting accuracy of 2D digital templating in total knee arthroplasty was the magnification factor, and a uniform and standardized procedure when taking the required radiographs for templating. Kniesel et al. used a calibration marker to adjust for magnification and were able to achieve accurate results of up to 100% [12]. Miller et al. also showed that the use of a calibration was more accurate than standardized magnification values [13]. Hernandez-Vaquero et al. did not use a calibration marker. By applying a constant and uniform distance between the X-ray tube and the knee, they could only achieve exact results in 50–55% [14]. In the current study, a calibration marker was always used and at a tolerance of a ± one size the accuracy was 96.5% for the femur and 94% for the tibia. Because many authors consider the accuracy of 2D templating to be insufficient, various size prediction strategies have been investigated. In a recent study, Wallace et al. compared 2D digital templating with a demography-based regression model in a prospective case series of 181 cases. With 2D templating, they achieved exact matches for the femoral component in only 35.4% and accurate matches in 86.2% of their cases. For the tibial component, exact matches were found in 36.5% and accurate in 85.1%. However, the protocol for acquiring radiographs was not uniform, as 53.6% of their radiographs did not use a reference marker. In those cases, they used a magnification factor of 115%. For the mathematical model, they reported exact matches in 43.7% and 43.7% and accurate matches in 90.1% and 95.6%, respectively [15]. When comparing manual and fully computer-automated templating and using conventional planning, Seaver et al. achieved exact matches in 56% and accurate matches in 41.6% on the femoral side in their cohort of 125 cases. On the tibial side, 61.6% exact and 33.6% accurate matches were achieved [16].

In light of recent advances in patient-specific implants and robot-assisted surgery, 3D computed tomography has been evaluated for templating in TKA. León-Muñoz et al. showed exact matches (± 0) in 95.8% for the femoral component and 92.6% for the tibial component and 100% accurate results in their cohort of 336 cases [17]. Pietrzak et al. examined 31 cases and found exact matches in 96.6% for the femoral and 93.1% for the tibial components for 3D templating as compared to 52.9% and 28.7%, respectively, for 2D templating [18]. Only one study was found that analyzed the feasibility of a 3D templating procedure without also using patient-specific instrumentation or robot-assisted surgery. Those authors showed 59% exact and 98% exact or accurate results in 3D templating as compared to 56% and 98%, respectively, for 2D templating [19]. Three-dimensional digital templating could lead to more accurate templating results, as was shown by patient-specific instrumentation and robot-assisted TKA. However, with these methods one must consider that the size of both the template and the bony resection, which is performed using patient-specific cutting guides or a robotic arm, is determined with the same algorithm. Therefore, the probability of a perfect match is logically greater.

As the number of different shapes as well as the incremental steps is increasing, there is also more need for an exact and reliable method with which to determine component size. Today, a plethora of different types of TKA implants exist, using ten to twelve femoral sizes (formerly five to six), inserts with 1 mm increments (instead of 2 mm) and side-specific tibial trays for many TKA systems (anatomical/asymmetrical shape). In total knee arthroplasty, templating accuracy is still not good enough to determine exact sizes preoperatively. In contrast to total hip arthroplasty where the interchangeable parts like inlay and head size are predetermined, in total knee arthroplasty the required height of the inlay is determined intraoperatively. This means that multiple inlay heights as well as PS and CR inlays must be readily available for at least three different sizes. This means the need for stock increases exponentially. A multitude of different components and surgical trays have to be close to the operating theater and must be held in stock at all times. This results in higher costs and more time needed for preparation and surgery. Accordingly, more accurate methods of implant size predictions need to be developed. The results of this study are consistent with results reported in the literature. When using 2D templating, exact matches can be expected in only approximately 50% of cases.

This study has several limitations, with the retrospective character probably being the most important one. Another problem entailed in this study was that total knee implants from two manufacturers were used; one manufacturer had eight size increments, the other ten. This may have led to a minor bias, but, as Fig. 1 shows, it did not have a serious effect on the results. With the exception of operative time, no economic indicator was evaluated. Although operative time was significantly longer for inaccurate templating as compared with exact match after correction for sex and adiposity (*p* = 0.028), total operating room time would provide more relevant information. The economic impact of accurate preoperative templating may even be more pronounced for patient-specific instrumentation [20].

A strength of this study is the large number of patients included. To our knowledge, this is the largest study of 2D templating in TKA. Furthermore, the data are not derived from artificial conditions in the context of a clinical trial, but reflect real-world conditions in patient care. In this study, 2D templating was highly standardized. All radiographs were taken with the same technique with a tube-to-film distance of 1.15 m and a radiopaque ball with a diameter of 25 mm serving as size reference. Templating was performed with a single well-validated templating program.

## Conclusions

The accuracy of 2D size templating in TKA is low, even in a specialized ENDOCERT arthroplasty center. The study findings challenge the usefulness of preoperative 2D size templating and highlight the importance of more reliable templating methods.

## Ethical approval

Ethical approval was obtained from the Ethics Committee of the Medical University of Innsbruck (reference number 1150/2019).

## Informed consent

All patients consented to publishing their data in anonymized form for scientific purposes.

## Abbreviations

TKA: Total knee arthroplasty
BMI: Body mass index
CR: Cruciate-retaining
PS: Posterior-stabilized
AP: Anterior–posterior
EPC: Endoprosthetics Center
EPCmax: Maximum Care Endoprosthetics Center
PACS: Picture Archiving and Communication System
